# Services for Mothers and Newborns During the Ebola Outbreak in Liberia: The Need for Improvement in Emergencies

**DOI:** 10.1371/currents.outbreaks.4ba318308719ac86fbef91f8e56cb66f

**Published:** 2015-04-16

**Authors:** Preetha Iyengar, Kate Kerber, Cuallah Jabbeh Howe, Bernice Dahn

**Affiliations:** Saving Newborn Lives, Save the Children, Washington, DC, USA; Saving Newborn Lives, Save the Children, Washington, DC, USA; Ministry of Health and Social Welfare; Ministry of Health and Social Welfare, Monrovia, Liberia

**Keywords:** ebola

## Abstract

Background: The magnitude of the Ebola outbreak in West Africa is unprecedented. Liberia, Guinea, and Sierra Leone are in the bottom ten countries in the Human Development Index, but all had made gains in child survival prior to the outbreak. With closure of healthcare facilities and the loss of health workers secondary to the outbreak, the region risks reversing survival gains achieved in maternal and newborn health. Methods: Anonymized service utilization data were downloaded from the Liberia District Health Information Software (DHIS) 2 for selected maternal health services at PHC facilities in Margibi and Bong Counties from March 2014, when the first case of Ebola was reported in Liberia, through December 2014. Absolute numbers are provided instead of percentage measures because of the lack of a population-based denominator. Results: Overall, the data show a decrease in absolute utilization from the start of the outbreak, followed by a slow recovery after October or November. In Bong County, totals were less than 14% of the peak numbers during the outbreak for number of antenatal visits and pregnant women receiving intermittent preventive treatment for malaria in pregnancy (IPTp). For total deliveries, utilization was less than 33% of the highest month. In Margibi County, during what now appears to be the height of the outbreak, numbers dropped to less than 9% of peak utilization for antenatal care visits and 4% for IPTp. Total health facility deliveries dropped to less than 9% of peak utilization. Conclusion: It is clear that Bong and Margibi Counties in Liberia experienced a large drop in utilization of maternal health care services during what now appears to be the peak of the Ebola outbreak. As the health of women and their babies is being promoted in the post-2015 sustainable development agenda, it is critical that the issue of maternal and newborn survival in humanitarian emergency settings, like the Ebola outbreak, is prioritized.

## Background

The ongoing Ebola outbreak in West Africa is unprecedented in magnitude, and has devastated the countries of Liberia, Sierra Leone and Guinea since early 2014. While Liberia, Guinea, and Sierra Leone are in the bottom ten countries in the Human Development Index, all three had made gains in child survival prior to the outbreak. Liberia in particular had reduced under-five mortality 71% since 1990 and achieved Millennium Development Goal 4, reducing child mortality by two thirds, three years before the end date of 2015.^1^ Although under-five mortality rates have fallen, the rate of progress for children in the first month of life – the neonatal period – has been much slower.^1^ Sierra Leone and Guinea are currently amongst the top ten countries with the highest neonatal mortality rates (NMR), and for much of the past two decades, Liberia’s NMR was also amongst the worst.^1^ These statistics have implications for the wider health system given that the care and survival of vulnerable newborn babies is a sensitive marker of health system response and performance.^2^

Liberia has made a concerted effort to address maternal and newborn survival through its 2013 “Accelerated Action Plan to Reduce Maternal and Newborn Mortality (AAP)”. The plan has five main objectives: 1) Increase the number and quality of skilled attendants for maternal and neonatal health services to staff health facilities with 24 hour care; 2) Increase coverage and access to quality comprehensive and basic emergency obstetric and neonatal care and essential maternal and newborn health care; 3) Increase access to and utilization of family planning services; 4) Expand and strengthen outreach and community-based services to improve coverage of maternal newborn health care; and 5) Improve management of maternal and newborn health services at national and county levels. The 2013 Liberia Demographic and Health Survey (DHS) showed that nationally 96% of women received at least one antenatal care visit, and that 61% of women were delivered by a skilled provider with almost all of these deliveries taking place in health facilities.^3^ To improve implementation of the AAP, a situation analysis of newborn health in Liberia and an assessment of barriers took place in 2013,^4^^,^ 5 however the Ebola outbreak brought both implementation and monitoring of this program to a halt.

The Ebola outbreak began in December 2013 in Guinea, spreading to Foya District in neighboring Liberia in March 2014, and to Sierra Leone in May 2014.^6^ In all three countries, the initial focus of the response focused on establishment of Ebola treatment centers, improving case reporting, and contact tracing.^6^ Despite these efforts, the outbreak continued to spread, complicated by weak healthcare infrastructure and community mistrust and resistance.^6^ The response from most of the international community only occurred months later in August 2014, when it was declared an international emergency by the World Health Organization (WHO).^7^ The outbreak has now directly affected over 25,000 people in West Africa, making it the largest outbreak in history. While the focus of the response was initially on isolation and treatment of Ebola patients, it soon became clear that the outbreak would have repercussions on the health of the general population, and that primary healthcare services needed to be restored.

In Liberia, all 15 administrative counties were affected by Ebola, accounting for approximately 40% of deaths in the outbreak.^8^ Loss of healthcare workers and fear in the community resulted in a closure of 62% of healthcare facilities by September 2014. Some counties that were heavily affected by the virus were forced to completely close all primary healthcare (PHC) facilities, thereby crippling an already fragile health care system. In 2011, Liberia had only 12.8 physicians, midwives and nurses per 10,000 people, far below the minimum WHO benchmark of 23 per 10,000.^9^ Now, over 175 Liberian health care workers have died from Ebola, leaving Liberians with even less access to healthcare services, and many mothers and newborns at risk of unnecessary complications during pregnancy, childbirth and the critical days thereafter.^8^ Pregnant women and their newborns are more vulnerable to Ebola than the general population, but there are additional risks even in women and newborns not directly infected. With closure of healthcare facilities and the loss of health workers, two major health system building blocks, the region risks reversing survival gains achieved in maternal and newborn health.

In Liberia, as part of the Ebola response in September and November 2014, Save the Children and other partners provided support to primary healthcare facilities in Margibi County (estimated population: 200,000) and Bong County (estimated population: 330,000).^10^ This support included training health facility staff in infection prevention and control, provision of essential health equipment and medications, and ongoing supportive supervision to ensure standards for health worker and patient safety were maintained. Before this support was widely available in Margibi, all of the 35 public and private health facilities were closed in September 2014, as healthcare facilities were unsafe for patients and health workers in light of the outbreak. In Bong, the 40 public and private health facilities remained open, but limited services were provided due to lack of health worker capacity and safety concerns. In this paper, we assess the impact of the Ebola outbreak on the utilization of selected maternal and newborn health services in health facilities in Bong and Margibi Counties in Liberia.

## Methods

Anonymized service utilization data were downloaded from the Liberia District Health Information Software (DHIS) 2 for selected maternal health services at PHC facilities in Margibi and Bong Counties from March 2014 to December 2014. The total number of women accessing antenatal care, intermittent preventive treatment for malaria in pregnancy (IPTp), and delivering at the facility was examined. These indicators were selected because they have an impact on both maternal and newborn health. Other indicators more specific to newborn health outcomes and care received (e.g. neonatal deaths, postnatal care) were only recently added to the DHIS2 and data are not yet consistently collected. Absolute numbers are provided instead of percentage measures because of the lack of a population-based denominator of all births occurring in the respective counties.

## Results

Overall, the data show a decrease in absolute utilization from the start of the outbreak, reaching a nadir in August or September, followed by a slow recovery after October or November in the number of total antenatal visits, total deliveries, and number of pregnant women receiving IPTp by county. In Bong County, where clinics remained open, there was a decline in utilization starting in March with a nadir in the month of August. In this month, the totals were less than 14% of the peak numbers for number of antenatal visits and pregnant women receiving IPTp (Figure 1, 2). For total health facility deliveries, utilization at the lowest point was less than 33% of the highest month (Figure 3). In Margibi County, where public clinics closed during what now appears to be the height of the outbreak, numbers dropped to less than 9% of peak utilization for antenatal care visits and 4% for IPTp (Figure 1, 2). Total health facility deliveries dropped to less than 9% of peak utilization in September (Figure 3).

**Total Number of Antenatal Care Visits in Bong and Margibi County Health Facilities:**
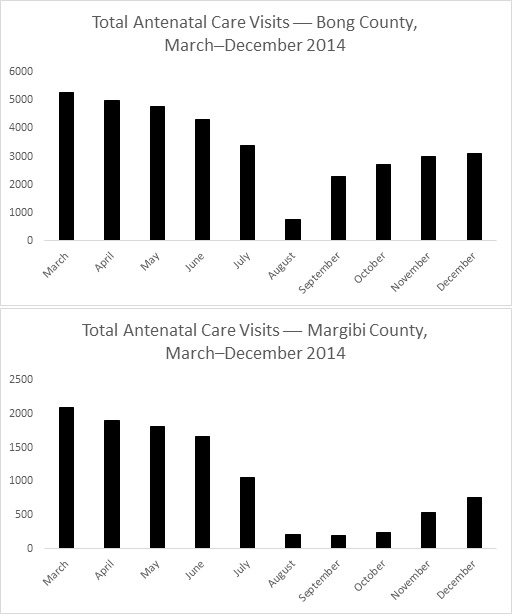

**Number of Pregnant Women Receiving Intermittent Preventive Treatment for Malaria (IPTp) in Bong and Margibi County Health Facilities:**
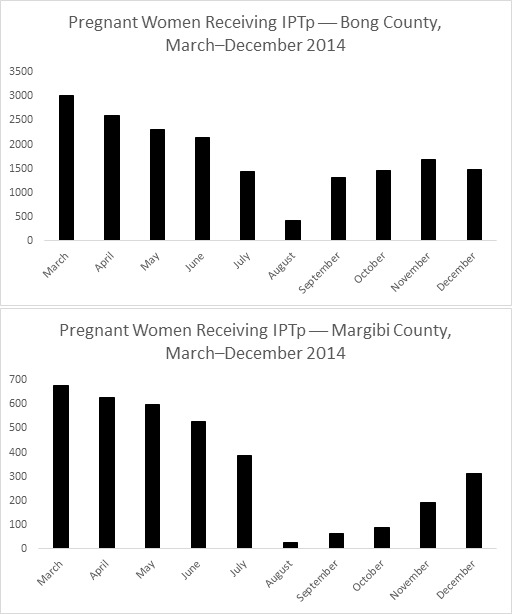

**Total Number of Deliveries in Bong and Margibi County Health Facilities:**
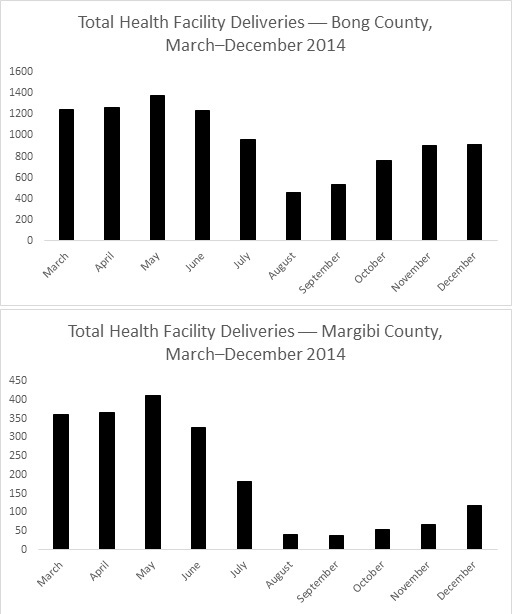

## Discussion and Conclusion

From the DHIS2 data, it is clear that Bong and Margibi Counties in Liberia experienced a large drop in utilization of maternal health care services during what now appears to be the height of the Ebola outbreak. Sadly, these drops correlate temporally with the peaks in infections amongst health workers in Bong and Margibi county health facilities.^11^ While the decrease could be due to lack of reporting, it is likely a reflection of a real change in usage in the context of disruption of healthcare services. The decline in care-seeking is likely due to both supply and demand factors modified by the outbreak, including the fear of contracting Ebola at health facilities and clinic closures. The outbreak progressed so quickly that access to necessary protective equipment and infection control measures were limited, making it evident to health officials and patients that crowds, particularly in health care settings, were a risk. Healthcare workers were both fearful of contracting the disease from sick community members presenting to their clinics and burdened by additional patient load, and therefore may have provided more limited services. In some cases, services were completely stopped to preserve healthcare worker safety. While this was recognized by the government and international partners, restoring services took months in the face of limited resources and logistical challenges. Based on data from previous humanitarian emergencies and the disruption of maternal health care in this crisis, it is likely that the neonatal mortality rate will have dramatically increased in the context of the outbreak.^12^ It is almost certain that many additional preventable stillbirths and newborn deaths will occur as a result of this emergency while the healthcare system is being restored.

There are several limitations to our analysis. While the DHIS2 data is reported from a large number of facilities, the reliability of the data has not been independently verified, and it is unclear if data was being collected and reported accurately. The correlation with health worker deaths could represent either a lack of data collection capacity at the time, or an actual change in service utilization. Either way, it reflects a decline in health system quality and highlights the importance of maintaining collection of healthcare data even during a time of crisis.

As health of women and their babies is being promoted in the post-2015 sustainable development agenda,^13^ it is critical that the issue of maternal and newborn survival in humanitarian emergency settings, like the Ebola outbreak, is prioritized. Newborn deaths now comprise 44% of deaths for children under 5 years of age; a critical gap that needs to be addressed in both the development and humanitarian sectors.^14^ It is necessary to address all aspects of healthcare that may be affected from the initiation of an emergency response, not just those related to the crisis, to end preventable deaths for mothers and newborns and maintain health system fidelity. Several cost-effective packages of care and interventions have been proven to reduce newborn deaths, including family planning, care for women during pregnancy, facility-based deliveries performed by equipped and well-supported skilled attendants, and integrated postnatal care for mother and newborn to promote healthy behaviors and address danger signs.^15^ The importance of having systems in place to monitor health data cannot be emphasized enough, so that critical gaps in care can be identified and addressed. The lessons learned from successful interventions to improve newborn health in the development context applied in emergency settings, both in preparedness and acute response, can help to improve maternal and newborn outcomes in emergencies in the future. As countries at highest risk for maternal and newborn deaths remain in Africa and in emergency contexts,^14^ it is imperative that primary health care is consistently addressed to give newborns and mothers, in any circumstance, a healthy start.

## Competing Interest

The authors have declared that no competing interests exist.

## References

[1] World Bank. Levels and Trends in Child Mortality : Estimates Developed by the UN Inter-Agency Group for Child Mortality Estimation (IGME) - Report 2014. Washington DC; 2014.

[2] Lam JO, Amsalu R, Kerber K, et al. Neonatal survival interventions in humanitarian emergencies: a survey of current practices and programs. Confl Health. 2012;6(1):2. 10.1186/1752-1505-6-2. PMC3488319 22824461

[3] Liberia Institute of Statistics and Geo-Information Services (LISGIS), Ministry of Health and Social, Welfare [Liberia], National AIDS Control Program [Liberia], ICF International. Liberia Demographic and Health Survey 2013. Monrovia, Liberia; 2014.

[4] Ministry of Health and Social Welfare. Situational Analysis of Newborn Health in Liberia. Monrovia, Liberia; 2013.

[5] Meyers N. Assessing the Implementation Barriers to the “Accelerated Action Plan to Reduce Maternal and Newborn Mortality” in Liberia. M.Sc. Thesis, Harvard School of Public Health. Boston, MA; 2013.

[6] Dixon MG, Schafer IJ. Ebola Viral Disease Outbreak — West Africa, 2014. Morb Mortal Wkly Rep. 2014; 63(25):548-549. PMC5779383 24964881

[7] World Health Organization. Statement on the 1st Meeting of the IHR Emergency Committee on the 2014 Ebola Outbreak in West Africa; 2014. http://www.who.int/mediacentre/news/statements/2014/ebola-20140808/en/.

[8] World Health Organization. Ebola Situation Report: 1 April 2015; 2015. http://apps.who.int/ebola/en/current-situation/ebola-situation-report.

[9] Downie R. The Road to Recovery: Rebuilding Liberia’s Health System. Washington DC; 2012.

[10] Republic of Liberia. 2008 NATIONAL POPULATION AND HOUSING CENSUS FINAL RESULTS.; 2009.

[11] Camacho A, Funk S, Carney J, Pollington T, Knight L. Visualisation of the MoHSW SitReps for Liberia and projections of the epidemic at the county level. 2014. http://ntncmch.github.io/ebola/liberia_sitrep.html#data-sources-extraction.

[12] Al Gasseer N, Dresden E, Keeney GB, Warren N. Status of women and infants in complex humanitarian emergencies. J Midwifery Womens Health. 2004;49(4 Suppl 1):7-13. doi:10.1016/j.jmwh.2004.05.001. 10.1016/j.jmwh.2004.05.001 15236698

[13] Mason E, McDougall L, Lawn JE, et al. From evidence to action to deliver a healthy start for the next generation. Lancet. 2014;384(9941):455-467. doi:10.1016/S0140-6736(14)60750-9. 10.1016/S0140-6736(14)60750-9 24853599

[14] Lawn JE, Blencowe H, Oza S, et al. Every Newborn: progress, priorities, and potential beyond survival. Lancet. 2014;384(9938):189-205. doi:10.1016/S0140-6736(14)60496-7. 10.1016/S0140-6736(14)60496-7 24853593

[15] Darmstadt GL, Bhutta ZA, Cousens S, et al. Evidence-based, cost-effective interventions : how many newborn babies can we save ? Lancet. 2005;365:19-30. 10.1016/S0140-6736(05)71088-6 15767001

